# Context processing as a moderator of the association between posttraumatic stress symptoms and the adaptation of maternal sensitivity following stress related to separation

**DOI:** 10.1002/imhj.70121

**Published:** 2026-10-05

**Authors:** Shannen Graf, Lara Detoledo, Sophie Wood, Dominik A. Moser, Sébastien Urben, Daniel S. Schechter

**Affiliations:** ^1^ Division of Child and Adolescent Psychiatry Department of Psychiatry Lausanne University Hospital and University of Lausanne Lausanne Switzerland; ^2^ Department of Child and Adolescent Psychiatry New York University Grossman School of Medicine New York New York USA

**Keywords:** context processing, hypervigilance, maternal sensitivity, posttraumatic stress

## Abstract

We aimed to assess the moderating role of context processing (i.e., context‐encoding and ‐memory) in the association between maternal posttraumatic stress symptoms (PTSS) and the changes in maternal sensitivity in reaction to a separation from their toddler. Thirty‐one mothers and their toddlers participated in the study in Switzerland. Mothers reported their current PTSS and performed a context processing task where they had to recognize contexts that they had previously seen paired with emotional faces. Maternal sensitivity was assessed during free play with their child and following a 3‐min separation and was coded using the CARE‐Index. A ratio score was calculated to assess the change in maternal sensitivity. Although the recognition of contexts previously associated with angry faces did not significantly moderate the relationship between maternal PTSS and the changes in maternal sensitivity, the recognition of contexts previously associated with neutral faces did. More PTSS led to a greater improvement of maternal sensitivity for mothers who had a better recognition of such contexts. This may be due to the ambiguity of neutral faces, which individuals with PTSS tend to focus on more closely, potentially due to their hypervigilance. Enhancing context processing may therefore help mothers use vigilance to improve sensitive caregiving.

## 1 INTRODUCTION

As much as 90% of the general population has been shown to have experienced at least one potentially traumatic experience according to the Diagnostic and Statistical Manual Fifth Edition (DSM‐5; American Psychiatric Association, 2013) criteria over the course of their lifetime (Kilpatrick et al., 2013). In the aftermath of such events, a minority of these individuals develop symptoms related to that event that cause distress and/or dysfunction, which phenomena are referred to as posttraumatic stress symptoms (PTSS; American Psychiatric Association, 2013). PTSS is divided into four clusters of symptoms: intrusion, avoidance, alterations in cognition and mood, and hyperarousal symptoms. PTSS can interfere with different aspects of daily functioning, such as sleep and emotion regulation, and may, thus, significantly impair one's quality of life (Zhao et al., 2023).

**Key points**

- Context recognition of neutral (but not angry) facial expressions significantly moderated the relationship between maternal PTSS and changes in maternal sensitivity.
- Mothers with higher PTSS who demonstrated better recognition of contexts previously associated with neutral faces showed greater improvements in sensitivity following a separation from their toddler.
- The results suggest that heightened vigilance (or hypervigilance) applied to ambiguous stimuli, such as neutral faces, may facilitate adaptive changes in maternal sensitivity during stressful situations.

**Statement of relevance to infant and early childhood mental health**

- This paper addresses how maternal posttraumatic stress symptoms (PTSS) interact with context processing to influence maternal sensitivity during a mother‐child separation, and by extension maternal caregiving behavior. It highlights the importance of maternal attention to contextual cues, especially ambiguous ones, and how this could help improve sensitivity, which has important effects on children at that age. These results suggest pathways for targeted early interventions using context processing to support sensitive caregiving in trauma‐affected mothers and families.

**Statement: Appreciation for diversity and anti‐racist approach**

- This study demonstrates an appreciation for diversity and an anti‐racist approach by recognizing how systemic inequities and researcher positionality influence mother‐toddler mental health research. We acknowledge that since the study included questionnaires in French, it limited the representativity of the sample and how these results apply to mothers who are either less literate or that do not speak French, including immigrant and/or racialized mothers whose experience may differ because of systemic inequities. The researcher's positionality carries privilege as it is embedded in a Western psychology's framework and influences several aspects, for example, how maternal sensitivity is defined and measured. This article aims to be mindful of these influences in the interpretation of the results. By examining mechanisms that may vary across individuals, the study acknowledges the role of sociocultural and systemic factors on mother‐child mental health and seeks to improve strategies that promote equality and inclusion in early caregiving research.

### 1.1 Motherhood and PTSS

PTSS affect not only the individuals who experience it but also their relationships with those around them, especially very young children who are highly dependent on their caregivers for physical needs, emotional regulation, and psychological security (Richter, 2004).

In the context of motherhood, this impact becomes even more relevant. Pregnancy and early parenthood can act as triggers for the reactivation or worsening of symptoms related to past potentially traumatic experiences through diverse mechanisms (Gordon et al., 2025). These include, for example, (re)traumatization during gynecological exams and childbirth (Gordon et al., 2025), as well as the reminiscence of memories linked to potentially traumatic events (Slade et al., 2009). The reactivation or worsening of PTSS can interfere with parenting during the early years, as PTSS is linked to more negative maternal representations of their relationship with their child (Schechter et al., 2005), more intense difficulties in attachment with the child (Erickson et al., 2019), as well as less maternal sensitivity (van Ee et al., 2016). This association is of particular concern because early childhood represents a critical phase in child development, when they rely most heavily on their parents (DePasquale & Gunnar, 2020).

Given this convergence, it becomes especially important to study how PTSS may be related to maternal behavior during this period. This is particularly relevant in moments of reactivation of stress, where the capacity for emotional regulation and attuned parenting can be most challenged. Understanding these dynamics is, thus, essential for developing interventions that support both maternal mental health and child development during this crucial period.

### 1.2 Maternal sensitivity

One of the aspects of a mother's caregiving behavior on which her PTSS has been suggested to have an effect is maternal sensitivity (MS; Lyons‐Ruth & Block, 1996), which is defined as being attentive to the signals of one's child, understanding them and responding to them in an appropriate manner (Ainsworth, 1969). However, mixed results have been reported, with some studies showed a decreased MS in mothers suffering from PTSS (Dozio et al., 2024; Schechter et al., 2010), and others showing no effect or even an increase in MS (Elansary et al., 2022; Hambleton et al., 2024). It is therefore likely that other factors influence this relationship.

A promising approach is to examine the changes of MS in reaction to stress. Brief mother–child separation is widely used as a stressor in early childhood research, as it activates attachment‐related distress and caregiving processes that are directly relevant to maternal sensitivity (Ainsworth et al., 2015). Studies have shown that in certain cases, the behavior's evolution might be more informative than a snapshot of behavior at a certain moment (Curley & Champagne, 2016; Madigan et al., 2016). Comparing MS before and after a stressful situation therefore gives finer information on how stress impacts MS. For example, two mothers might show a similar level of sensitivity in a non‐stressful situation, yet they could react differently to a stressful situation with one becoming less sensitive and the other staying the same or even becoming more sensitive. The reverse pattern (i.e., two mothers having the same level of sensitivity after a stressful situation while differing beforehand) is also possible. Taking into account such changes can therefore reveal differences in the way mothers regulate their sensitivity under stress, offering a more nuanced understanding of how MS fluctuates in relation to contextual factors, highlighting its dynamic and situation‐dependent nature.

### 1.3 Context processing

Context processing (or context‐encoding and ‐memory; CEM) may help clarify how PTSS affects MS. It refers to the ability to take the context into account when interpreting stimuli in their environment (Maren et al., 2013). It plays a central role in distinguishing threats from non‐threats and therefore reacting accordingly (Lambert et al., 2017). A comprehensive review in adult samples highlighted the relevance of this mechanism for the interpretation of faces specifically, especially for neutral faces, which are typically perceived as ambiguous and for which the viewer needs more information to understand the person's feelings and intentions (Wieser & Brosch, 2012).

Context processing seems especially relevant to the framework of parenting. Parenting involves the integration of multiple sources of information, including contextual information, to interpret their children's cues and react appropriately (Miller, 1995). The same behavior might indeed hint at different emotional states depending on the context in which it occurs: withdrawal might come from tiredness, but also wariness, and the context is of utmost importance to interpret signals correctly. Parents with stronger context processing abilities, meaning that they can more rapidly integrate a wider range of contextual cues, may therefore be better equipped to understand their children's signals within the contextual frame, supporting more attuned responses.

In addition, context processing abilities in the case of parenting may contribute to the parent's own emotional state during interactions with their child. By attributing ambiguous child behaviors to contextual elements, including the child's own mental states, instead of seeing those behaviors as inherently threatening, parents may better regulate their own emotions and react more appropriately. This is especially the case for mothers suffering from PTSS, as they are more disposed to interpreting ambiguous stimuli as threatening (Bomyea et al., 2017). Stronger context processing capacities may be especially beneficial for them, allowing them to differentiate between threatening and non‐threatening situations and thereby helping preserve their sensitivity.

Finally, as PTSS refers to a constellation of symptoms, one that might be more strongly associated with context processing is hypervigilance, defined as increased attention to one's surroundings. Since individuals who suffer from PTSS typically perceive the world as a dangerous place and are constantly searching for a potential threat, they are therefore excessively fearful about—and attentive to—their environment. Indeed, context processing of threatening faces is known to be hindered in individuals suffering from PTSS, due to heightened vigilance to and difficulty disengaging from threatening cues (Lambert et al., 2017). Indeed, the exploration of a moderating role of context processing is particularly important to test since some trauma experts have posited that context‐encoding and ‐memory difficulties may be central to the pathogenesis of PTSS risk intergenerationally (Liberzon & Abelson, 2016). It might thus also be beneficial to evaluate how the presence of threatening cues, in addition to ambiguous ones, interact with PTSS to affect parenting.

Moreover, the role of context processing is likely to differ depending on whether the cues are ambiguous or explicitly threatening. Ambiguous stimuli, such as neutral faces, do not convey clear indications on their emotional valence and therefore require contextual information to be interpreted accurately. Context processing, in that case, is essential to understand the stimulus, and deficits or biases in that process, such as the tendency to perceive stimuli as more threatening, which is characteristic of PTSS, might hinder contextualization and provoke maladaptive responses. In contrast, threatening stimuli convey a clear emotional message, without the need for much contextual information for interpretation. In such situations, context processing might rather support the regulation and contextualization of threat rather than its direct interpretation.

Given these conceptually distinct mechanisms, examining context processing in relation to both ambiguous and threatening cues separately may provide a more precise understanding of how PTSS relates to MS and why inconsistent results have been found.

Taken all together, these elements show the importance of the way individuals process contextual information, especially in the case of parenting, which seems like an interesting lead to fully understand the link between PTSS and MS. Mothers’ PTSS may indeed have a differential link to their sensitivity depending on their ability to process contextual information. In that sense, further studies integrating PTSS, context processing and MS would be valuable to better understand their dynamics, especially as they have been scarcely studied together in the literature to date.

### 1.4 The current study

To summarize, the vast majority of people experience potentially traumatic events, and some develop PTSS as a result. For mothers, PTSS can affect their caregiving, particularly in early childhood when toddlers rely on caregivers’ psychological availability for healthy social‐emotional development. However, the relationship between PTSS and MS remains unclear, with studies showing inconsistent results (Dozio et al., 2024; Elansary et al., 2022; Hambleton et al., 2024). One cognitive mechanism that might help explain these inconsistent associations is context processing. Better context processing capacities of ambiguous and/or threatening stimuli support more accurate interpretation of child cues and may thus lower the impact of PTSS on MS. It had, indeed, been shown in an earlier study of the same sample to modulate the relationship between maternal PTSS and mother‐reported child dysregulation, with stronger context processing abilities attenuating the positive link between PTSS and child dysregulation to a non‐significant level, suggesting its potential link to. Thus, understanding the interaction between PTSS, context processing, and the change in MS can shed light on their complex dynamics as well as the contradictory results found in the literature, and guide interventions to target better context processing in order to support sensitive caregiving in trauma‐affected mothers and families. For example, interventions that use videofeedback, with therapist‐parent joint attention to specific interactions within a controlled context in a clinical setting, may be especially useful. Such approaches can serve as a form of clinician‐guided exposure to relational situations that activate the mother's posttraumatic stress, while the therapist supports both emotion regulation and parental reflective functioning (e.g., Willheim & Schechter, 2025).

The aim of this pilot study is to assess the potential moderating role of context processing in the association between maternal PTSS and changes in MS in reaction to a stressful situation (i.e., separation) with their 1‐ to 3‐year‐old child. Specifically, we explored how performance in a context processing task involving context recognition moderates the link between maternal PTSS and changes in MS, depending on the emotion displayed by a face presented at the same time as the context (i.e., anger or neutral). We hypothesized that context processing would moderate this relationship, both in the case of ambiguous and threatening faces, with better context processing abilities attenuating an otherwise negative link between PTSS and the change in MS.

## 2 METHODS

### 2.1 Sample

Thirty‐one mothers and their 12‐ to 36‐month‐old child participated in the study between December 2020 and November 2021. They were recruited through advertisements in Lausanne University Hospital and on social media (e.g., Facebook, LinkedIn). Participants’ written consent was obtained after they received oral and written information. Inclusion criteria included being between 18 and 45 years old, having a 12–36‐month‐old biological child with whom they had lived for the majority of the child's life, speaking French, not having an active psychosis or substance abuse, and not having any handicap that could prevent participation in the study. Demographic information of the sample can be found in Table 1. The study's procedure was approved by the Research Ethics Committee (Vaud, #2019‐02490).

**TABLE 1:** Demographics.

Variable	Mean ± SD	%
Maternal age	33.87 ± 4.14 y.o.	
Child age	22.66 ± 7.01 m.o.	
Child sex		
Girls		41.9%
Boys		58.1%
Mothers have other children		
Yes		51.6%
No		48.4%
Nationality		
Switzerland		64.5%
Europe		32.3%
Outside Europe		3.2%
Family gross annual income in CHF		
Below 25,000 CHF		6.5%
25,000–49,999 CHF		6.5%
50,000–74,900 CHF		16.1%
75,000–99,999 CHF		12.9%
100,000–149,999 CHF		38.7%
150,000–199,999 CHF		12.9%
200,000 CHF or more		6.5%

Abbreviations: CHF, Swiss francs; m.o., months old; y.o., years old.

### 2.2 Procedure

Participants completed the Posttraumatic Stress Disorder Checklist for DSM‐5 (PCL‐5; Blevins et al., 2015) online to evaluate their current PTSS. They also performed a context processing (or CEM) task during a one‐hour appointment in a research office. Finally, MS was assessed using the CARE‐Index (Crittenden, 1979–2004) during free play with their child as well as after a 3‐min separation.

### 2.3 Measures

#### 2.3.1 Maternal PTSS

Maternal current PTSS were assessed online on REDCap using the PCL‐5 (Blevins et al., 2015). It is composed of 20 items evaluating posttraumatic stress symptomatology in the last month in relation to the event that they consider the most traumatizing in their life. Scoring is kept continuous in order to evaluate the severity of their current level of PTSS on a spectrum.

#### 2.3.2 Context processing

Context processing was evaluated using a CEM task from Lambert et al. (2017) and carried out on E‐Prime 2.0, which included both an encoding phase and a recognition phase. For the encoding phase, the exact same task as Lambert et al. (2017) was used (see article for more information). Participants saw a face embedded into a contextual background picture (2000 ms), then a fixation cross (1000–5000 ms), and then another picture of a face without a contextual background this time (2000 ms). When they saw the latter, they were instructed to respond as quickly and accurately as possible if the face was the same one (i.e., same person displaying the same emotion) as the one presented with a contextual background at the beginning of the trial, using two keyboard keys (1 = identical, 0 = different). They did not receive any feedback after each answer. The instructions intentionally left out anything regarding the contextual backgrounds to minimize awareness of the contexts. For the recognition phase, of which participants were not aware, they saw contextual backgrounds without a face and had to determine, as quickly and accurately as possible, if they had seen that context during the encoding phase or not. Lambert et al. (2017)’s recognition phase consisted of face‐context combination, and participants were asked to indicate if they had seen that combination during the encoding phase. However, since the focus of our study was performance in the recognition of *contexts*, we adapted the task to have the best indicator of context encoding. The score used is the Balanced Integration Score (BIS; Liesefeld & Janczyk, 2019) and takes into account both reaction time and accuracy. The quicker and more accurate the response is, the higher the score is.

The faces in the encoding phase were either angry, happy, or neutral. For the purpose of this paper, we focused on the recognition of contexts that were associated with angry faces and neutral faces, as they best represented potentially challenging emotions. They are referred to as anger‐associated contexts and neutral‐associated contexts, respectively.

#### 2.3.3 Maternal sensitivity

MS was derived from video excerpts of a mother‐child interaction following the Modified Crowell Procedure (Schechter et al., 2010; Zeanah et al., 2000). Participants were aware and agreed that the interaction was filmed. Every mother‐child dyad was asked to play freely in a room with a basket full of toys, in the absence of the research assistant. After 8 min, the research assistant knocked on the door, indicating to the mother that she had to leave the room. Her child stayed in the room and was constantly monitored by another research assistant who was watching live from a nearby room. The separation lasted 3 min, or less if the mother asked to go back into the room earlier. She then re‐entered, and their reunion was filmed for 2 min.

Interactions were coded using the Toddler CARE‐Index (Crittenden, 1979–2004, 2006; Kunster et al., 2010). As only 3 children were under 16 months old, the Toddler version was used for all children for consistency. MS was assessed on a scale ranging from 1 to 7 for both the free play and the reunion. Two certified raters coded all interactions and achieved excellent inter‐rater reliability (ICC = .93). A ratio score was then calculated to determine the increase of MS during the reunion compared to the free play, computed as follows: (MS during reunion—MS during free play)/MS during free play. The goal with using this score was to see how the mother would adapt her sensitivity to her child following a stressful situation (i.e., separation). Video was not obtained for one dyad due to technical issues.

### 2.4 Data analysis

All analyses were performed using Jamovi 2.3.21. Since the data distribution did not follow Gaussian‐like distribution, coefficients of Spearman (*ρ*) correlations were calculated between the variables of interest as well as potential control variables, namely MS during free play, separation time, child age, and sibling position. Then, two models were tested: one to assess the moderating role of anger‐associated contexts on the association between maternal current PTSS and the adjustment of MS following the separation, and the other to assess the moderating role of neutral‐associated contexts on the same association. For both models, MS at free play was used as a control variable, as well as separation time, as they can both influence the change in sensitivity. Since socioeconomic status did not significantly correlate with any of the variables, we did not include it in the models to preserve statistical power.

Finally, we conducted highly exploratory analyses by replacing maternal PTSS score by their score on an item measuring hypervigilance in the moderation models, in order to isolate this variable's effects. These analyses should be interpreted with caution, as they rely on a single item, which limits reliability and construct validity, and the findings must therefore be considered highly tentative and are not intended to support firm conclusions. The power analysis (performed with G*Power 3.1) for regression models with five predictors, *α* < .05, and 31 mother‐child dyads revealed an achieved power of .89 to observe a large effect size (chosen for clinical relevance).

## 3 RESULTS

Table 2 reports descriptive data. According to their PTSS score, 4 mothers out of 31 (7.75%) met the criteria for potential posttraumatic stress disorder (PTSD, score ≥ 31), with 4 more having a subthreshold score indicative of significant symptoms (between 26 and 30). Seven mothers reported no PTSS.

**TABLE 2:** Descriptives.

Variable	Mean ± SD	Range
Maternal PTSS	15.13 ± 14.45	0 to 62
Recognition of neutral‐associated contexts	.00 ± 1.51	−4.46 to 2.21
Recognition of anger‐associated contexts	.00 ± 1.08	−2.57 to 1.95
Changes in maternal sensitivity (ratio)	−.10 ± .36	−.67 to 1.00
Maternal sensitivity at free play	5.43 ± 1.72	2 to 7
Separation time (s)	144.26 ± 56.79	0 to 180
Child age (months)	22.66 ± 7.01	11.24 to 35.71

Abbreviation: PTSS, posttraumatic stress symptoms.

### 3.1 Correlations

PTSS and the change in MS between free play and reunion were not significantly correlated (*ρ *= .254, *p =* .183). The performance in the recognition of anger‐associated contexts showed a trend‐level correlation (i.e., .05 < *p* < .10) with performance on the recognition of neutral‐associated contexts (*ρ* = .317, *p* = .083). Separation time (i.e., how long the mother stayed out of the room) also showed a trend‐level correlation with the change in sensitivity following separation (*ρ *= .354, *p =* .055). Since the child's age was not significantly associated with any of the variables, it was not added as a control variable in our models to preserve statistical power.

### 3.2 Regression models

Table 3 reports the results of the regression models.

**TABLE 3:** Regression model—Change in maternal sensitivity (neutral‐associated contexts).

Predictor	Estimate (Std. error)	*p*
Recognition of neutral‐associated contexts	−.11 (.07)	.147
Maternal PTSS	.01 (.00)	.122
Sensitivity at free play	.03 (.03)	.400
Separation time	.00 (.00)	.155
Maternal PTSS × recognition of neutral‐associated contexts	.01 (.00)	.011^*^

Abbreviation: PTSS, posttraumatic stress symptoms.

*p < .05.

The moderation model using performance on the recognition of anger‐associated contexts as the moderating variable for the association between maternal current PTSS and the change in MS after a separation with the child was not significant (*F*(5, 24) = 2.02, *p* = .112).

The moderation model using performance on the recognition of neutral‐associated contexts as the moderating variable on the association between maternal current PTSS and the change in MS after separation was significant (*F*(5, 24) = 4.30, *p* = .006, *R* ^2^ = .472). In particular, the interaction between maternal current PTSS and performance on the recognition of neutral‐associated contexts was significant (*β* = .01, *p* = .011, 95% CI [.002, .017]). The better the performance on the recognition of neutral‐associated contexts, the stronger the association between current PTSS and greater MS following separation is. More precisely, current maternal PTSS appears not to have an impact on the change in MS for mothers with a low recognition of neutral‐associated contexts (*β* = −.004, *p* = .530), but has a positive impact (i.e., increase) on the change in MS for those with a high recognition of these contexts (*β* = .016, *p* = .009; Figure 1). A Johnson‐Neyman analysis indicated that the link between current PTSS and changes in MS was significant for values of recognition of neutral‐associated contexts below −3.764 and over .161. The lower part included 3.2% of participants (*n* = 1), whereas the upper significant region included 54.8% of participants (*n* = 17), suggesting that the interaction effect was mostly driven by the upper values.

**FIGURE 1:**
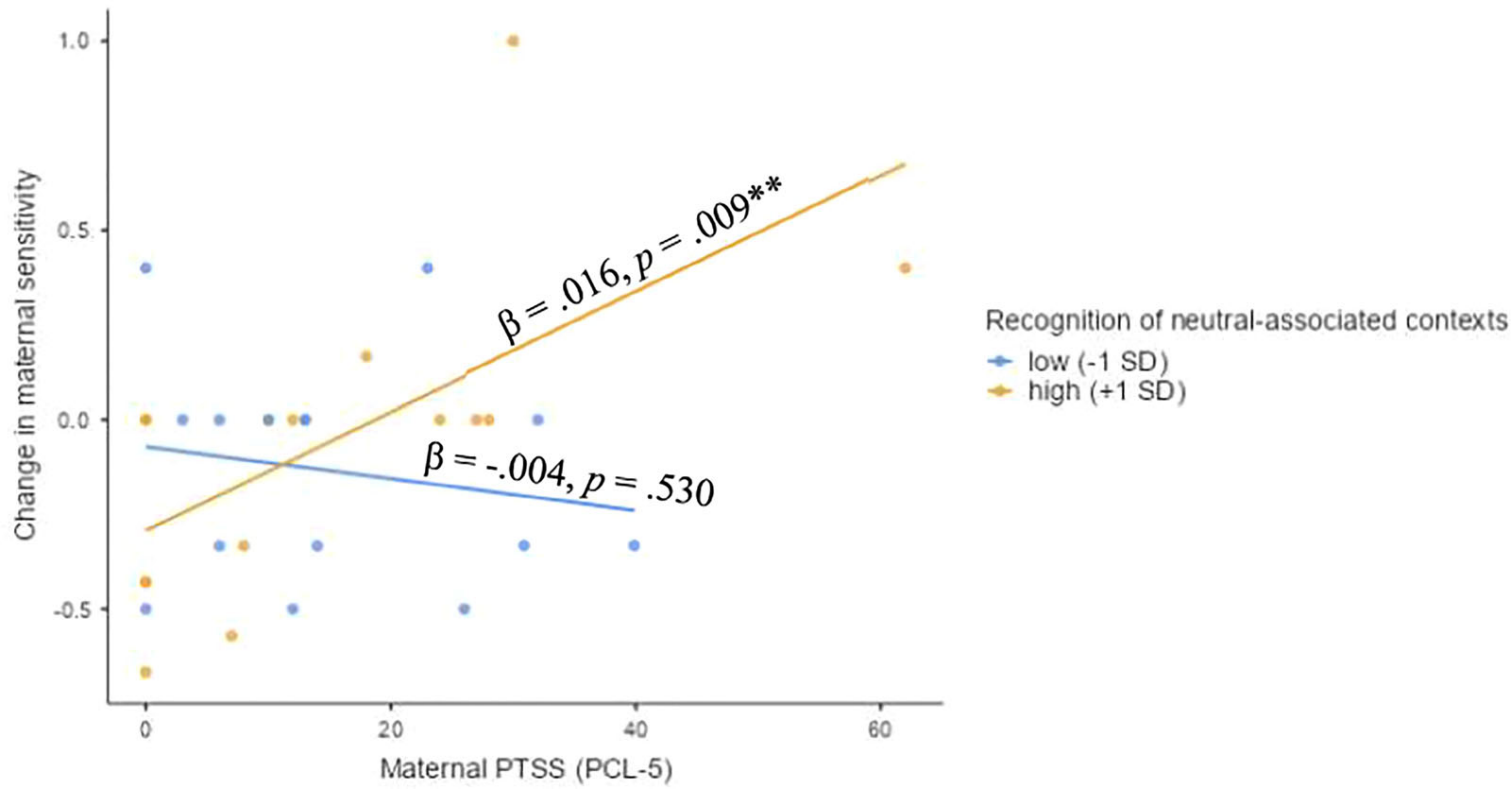
Moderating effect of context processing (neutral‐associated contexts) on the relationship between maternal posttraumatic stress symptoms and the change in maternal sensitivity. This figure shows that higher context processing of neutral‐associated contexts is associated with a greater relationship between PTSS and changes in maternal sensitivity, whereas lower context processing does not have an impact on the relationship. PCL‐5, Posttraumatic Stress Disorder Checklist for DSM‐5; PTSS, posttraumatic stress symptoms; SD, standard deviation.

### 3.3 Exploratory analyses

To investigate further these associations, we conducted post hoc analyses using the same moderation models but replacing PTSS with a specific item evaluating hypervigilance. The moderation model using performance on the recognition of anger‐associated contexts as a moderator of the association between the hypervigilance item and the change in MS was nearly significant (*F*(5, 24) = 2.61, *p* = .051, *R* ^2^ = .352). The interaction between the hypervigilance item and performance on the recognition of anger‐associated context was significant (*β* = .13, *p* = .018, 95% CI [.025, .239]), as was separation time (*β* = .002, *p* = .045, 95% CI [.000, .005]). This time again, the better the performance on the recognition of anger‐associated contexts, the more positive impact hypervigilance had on the increase of MS. However, hypervigilance appears not to have an impact on the change in MS for mothers with both a low recognition of anger‐associated contexts (*β* = −.062, *p* = .455) and for those with a high recognition of these contexts (*β* = .113, *p* = .087; Figure 2). A Johnson‐Neyman analysis indicated that the link between the hypervigilance item and changes in MS was significant for values of recognition of anger‐associated contexts below −2.68 and over .475. The lower significant region did not include any participant, as the significant threshold was slightly below the minimum observed value, whereas the upper significant region included 38.7% of participants (*n* = 12), suggesting that the interaction effect was solely observed in the upper values (i.e., participants with higher recognition of anger‐associated contexts).

**FIGURE 2:**
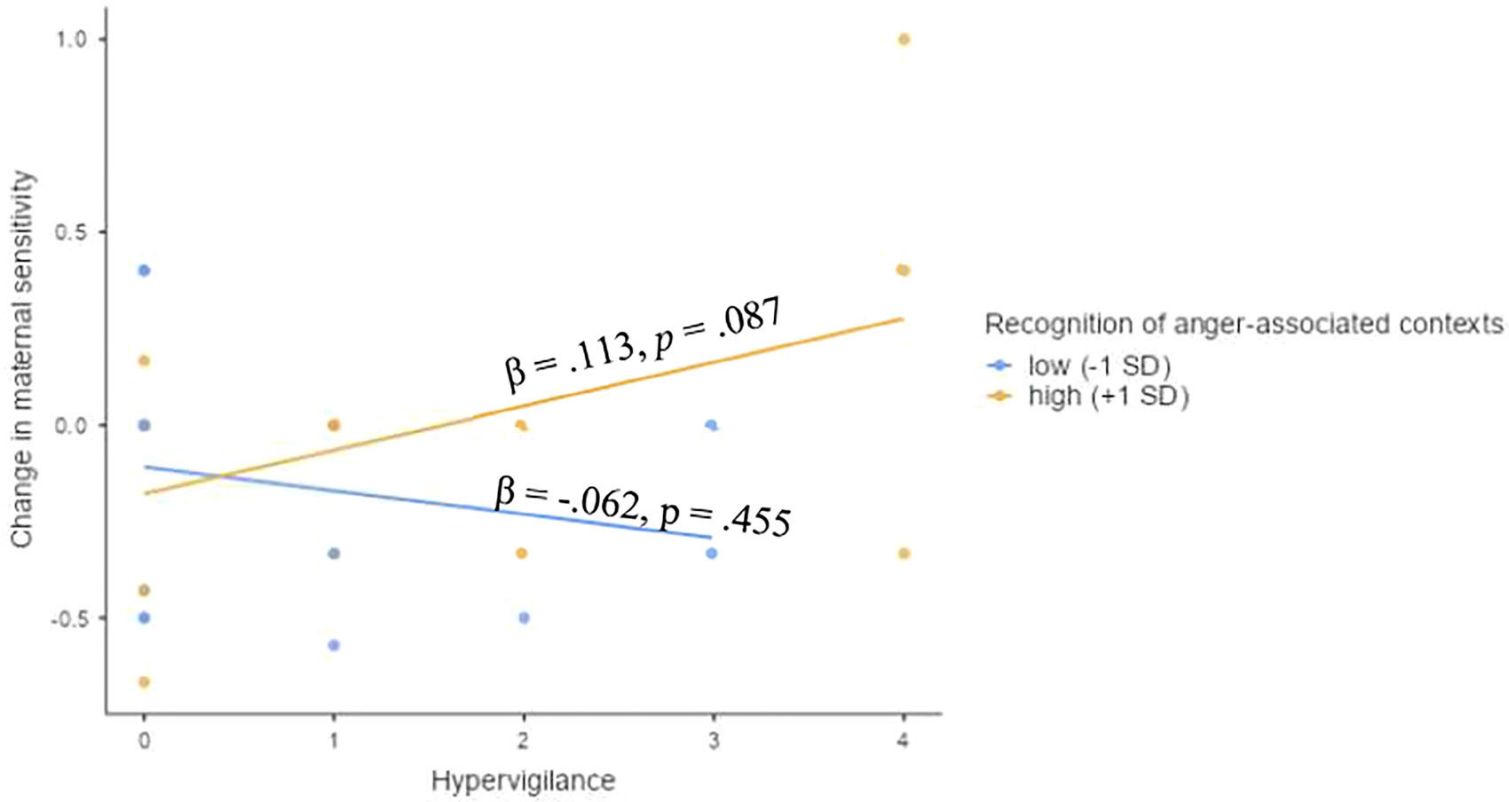
Moderating effect of context processing (anger‐associated contexts) on the relationship between hypervigilance and the change in maternal sensitivity. This figure shows interaction lines indicating that there is a moderation by context processing of anger‐associated contexts on the relationship between hypervigilance and the change in maternal sensitivity, but that neither higher context processing nor lower context processing is associated with a greater or weaker relationship between PTSS and changes in maternal sensitivity. SD, standard deviation.

The moderation model using performance on the recognition of neutral‐associated contexts as a moderating variable of the association between the hypervigilance item and the change in MS was significant (*F*(5, 24) = 3.96, *p* = .009, *R* ^2^ = .452). The interaction between the hypervigilance item and the performance on the recognition of neutral‐associated context was significant in the model (*β* = .098, *p* = .004, 95% CI [.034, .162]). The better the performance on the recognition of neutral‐associated contexts, the more positive impact hypervigilance had on the increase of MS. Hypervigilance appears not to have an impact on the change in MS for mothers with a low recognition of neutral‐associated contexts (*β* = −.094, *p* = .133), but has a positive impact on the change in MS for those with a high recognition of these contexts (*β* = .185, *p* = .008; Figure 3). A Johnson‐Neyman analysis indicated that the link between the hypervigilance item and changes in MS was significant for values of recognition of neutral‐associated contexts below −1.945 and over .664. The lower significant region included 9.7% of participants (*n* = 3), whereas the upper significant region included 35.5% of participants (*n* = 11), suggesting that the interaction effect was primarily observed in the upper values.

**FIGURE 3:**
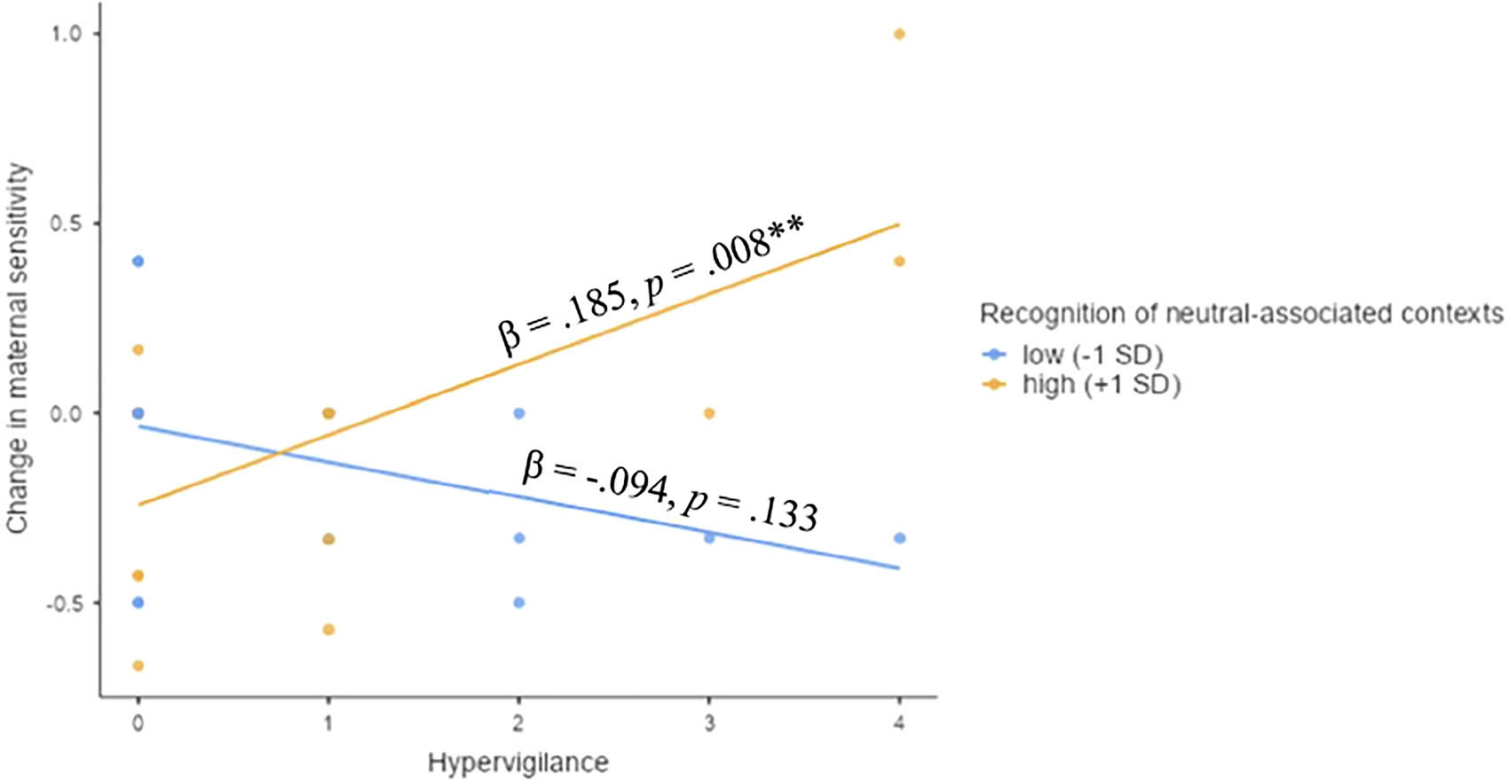
Moderating effect of context processing (neutral‐associated contexts) on the relationship between hypervigilance (1 item) and the change in maternal sensitivity. This figure shows interaction lines indicating that higher context processing of neutral‐associated contexts is associated with a greater relationship between hypervigilance and changes in maternal sensitivity, whereas lower context processing does not have an impact on the relationship. SD, standard deviation.

## 4 DISCUSSION

The aim of this study was to assess the moderating role of context processing in the association between maternal PTSS and the adaptation of MS after a stressful situation (i.e., mother‐child separation) for toddlers and mothers. As our sample primarily includes mothers with relatively low PTSS that are socioeconomically rather advantaged, the following interpretation should be understood as applying specifically to subclinical PTSS and may not extend to clinical populations.

Our results showed that maternal PTSS were not significantly associated with the changes in MS following a mother's separation from the child. However, we found that while the recognition of neutral‐associated contexts moderated the relationship between maternal current PTSS and the change in MS (i.e., increase), the recognition of anger‐associated contexts did not do so. More precisely, for mothers with a better recognition of neutral‐associated contexts, more maternal current PTSS was associated with a greater increase in MS, but this was not the case for anger‐associated contexts. We hypothesized that context processing would moderate this relationship in both contexts, with better context processing abilities attenuating an otherwise significant link between PTSS and a decrease in MS. This hypothesis was therefore only partially supported. Specifically, only context processing related to neutral‐associated contexts moderated this relationship, and greater context processing skills were associated with a significant positive link between PTSS and an increase in MS, which was otherwise non‐significant.

First, our findings suggest differences depending on facial emotion. Neutral facial expressions provide limited information about emotional valence and are rather perceived as ambiguous, thereby placing greater demands on contextual interpretation. In contrast, angry facial expressions convey clear and salient emotional signals that can be rapidly interpreted with relatively little reliance on contextual cues. Thus, our results suggest that, in this context, processing contextual information to interpret the valence of an ambiguous facial expression is more critical than using contextual cues to explain the presence of a clearly angry facial expression.

This finding contributes to the ongoing debate in the literature about the relationship between PTSS and MS (Dozio et al., 2024; Elansary et al., 2022; Hambleton et al., 2024). It appears that the association between PTSS and MS may depend on the mother's ability to interpret the context of certain situations. Specifically, when ambiguous stimuli are present, mothers with more severe PTSS but stronger context processing abilities may exhibit greater sensitivity following stress. This finding indicates that PTSS do not uniformly impair caregiving and that under certain conditions, it may be associated with more attuned maternal responses.

This may be explained by the Adaptive Calibration Model (Del Giudice et al., 2011), which suggests that exposure to adversity can lead to differences in individuals’ stress systems. For some, having to adapt to such challenges may result in more positive ways of dealing with stress, which can create more resilience and support more beneficial adaptations to adverse conditions across the lifespan. Within this perspective, mothers who, despite their higher levels of PTSS, have better context processing capacities in response to unclear stimuli may be the ones who have managed to successfully adapt to adverse conditions, and their sensitivity is therefore enhanced, rather than worsened or merely preserved. This may not be the case with clinical populations, where higher symptom severity and chronic stress may constrain adaptive regulatory processes, but further research is needed.

Within that perspective, context‐sensitive vigilance that is preserved despite the presence of higher levels of PTSS may be even more beneficial. This interpretation is supported by Carney et al. (2025), who found that greater exposure to violence was associated with better child regulatory capacities among mothers with higher resilience. Similarly, in our sample, preserved vigilance in contexts with ambiguous stimuli among mothers with higher PTSS may reflect a form of resilience, where increased vigilance is directed toward caregiving demands rather than generalized threat detection. This aligns with the nature of the study, as the mothers knew that only their child was in the room and that the child's safety was guaranteed by the research assistants who were looking at the child via cameras. While recognition of anger‐associated contexts is important in other situations (Lambert et al., 2017; Graf et al., 2025), our results suggest that it plays a less significant role in situations where the threat is not perceived as immediate.

Thus, our results suggest that this increased vigilance associated with PTSS can have a positive impact on an increase in MS between free play and the reunion after a separation, but this effect is contingent on the mother's ability to process contextual cues in the presence of an ambiguous stimulus. It seems to be the case especially if this increased vigilance is also applied to contextual cues in the case of an unclear situation.

Our post hoc highly exploratory analyses, which used an item assessing hypervigilance as an indicator of PTSS, supported our initial interpretation and provided additional nuance by suggesting hypervigilance to be a contributing mechanism. When hypervigilance was examined as a specific PTSS symptom, the model incorporating neutral‐associated contexts as the moderating variable between hypervigilance and change in MS reached significance, which was consistent with our primary results. However, the model using anger‐associated contexts as the moderator also approached significance, a pattern which was not observed when using the total PTSS score, indicating that hypervigilance may also interact with other emotional cues in ways that are more specific than the broader PTSS construct. These findings suggest that hypervigilance may partly account for the observed moderation while also suggesting that specific PTSS symptoms and symptom clusters (i.e., hyperarousal in this instance) may interact with contextual cues in more nuanced ways.

Rather than being contrasting with Lambert et al. (2017)’s study on children and adolescents, these findings complement their results by adding that in adulthood and caregiving, specific symptoms may influence context behavior and processing differently. However, the exploratory nature of these analyses underscores the need for further research examining distinct PTSS profiles and their differential contributions to parenting behavior.

Taken together, the present findings underscore the importance of context processing in ambiguous situations for understanding when and how PTSS relate to changes in MS. They suggest that, for mothers with PTSS, heightened vigilance to contextual cues may be associated with more sensitive caregiving, which may be a sign of a positive adaptation to adverse conditions. Context processing may especially interact with hypervigilance, one of the symptoms of PTSS, but these results are highly exploratory and must be replicated.

From a clinical perspective, these findings suggest that videofeedback interventions may be particularly beneficial, especially when they focus on ambiguous or neutral interactional moments. Such approaches, in which the therapist supports both regulation and reflective functioning, can help parents better interpret the physical, emotional, and developmental context of interactions. Our results seem to indicate that supporting mothers in making sense of subtle or unclear cues may be especially important, as it may allow trauma‐related vigilance to be directed toward more adaptive, sensitive caregiving during stressful interactions.

### 4.1 Limitations

The first limitation in our pilot study is the rather small sample size, which may constrain the generalizability of our findings, despite adequate statistical power. Additionally, the levels of PTSS in this sample are relatively low, potentially limiting the applicability of our results to clinical populations. Future research should replicate these analyses with a larger and more symptomatic sample to determine whether the observed associations hold in different contexts.

Additionally, context processing may be linked to broader cognitive processes, such as memory and intelligence, which were not measured in this study. It could be interesting to replicate our results while controlling for these variables, in a bigger sample to guarantee enough statistical power.

Although the CARE‐Index has been used across a range of cultural environments, it was designed in a specific cultural environment and therefore still has limitations related to accurately capturing sensitivity in non‐Western contexts (Crittenden et al., 2005). Cultural differences still shape how sensitivity is expressed (Crittenden et al., 2005; Prag et al., 2025), and these differences may not be fully captured by the CARE‐Index coding system. As a result, its application to diverse cultural groups may reduce the ecological validity of the findings, and this constitutes a limitation of this study.

Another limitation is that only maternal indices, and not child indices, were coded during the interaction. Consequently, we were unable to account for the impact of individual differences in child's behavior on the mother, despite evidence suggesting that bidirectional influences are integral to dyadic interactions. Future studies should incorporate both maternal and child observational indices to provide a more comprehensive understanding of these dynamics.

Finally, due to the study's cross‐sectional design, we cannot infer prediction or temporal changes. Conclusions about changes over time cannot therefore be drawn from this sample, and findings should be interpreted as correlational rather than predictive. This underscores the need for longitudinal designs that permit the observation of such effects.

## 5 CONCLUSION

While the association between PTSS and MS has been the subject of debate in the literature, the results of the present study suggest that conflicting results may be, at least in part, related to the mother's ability to take contextual cues into account. In sum, if mothers display better context processing when faced with an ambiguous or unclear stimulus, their heightened vigilance, which is characteristic of their PTSS can have a positive impact on their sensitivity to the child's following an important stressor such as that of mother‐child separation in an unfamiliar environment for a toddler. This suggests that interventions aimed at enhancing context processing, particularly of apparently neutral, ambiguous contexts, may help mothers leverage their heightened vigilance to increase their sensitive responsiveness to their child during stressful interactions.

## CONFLICT OF INTEREST STATEMENT

The authors declare no conflicts of interest.

## USE OF ARTIFICIAL INTELLIGENCE

Artificial intelligence (AI) tools (i.e., ChatGPT and DeepL) were used solely to assist with language refinement and editing, and not to generate content. All content was produced by the authors, including but not limited to ideas, analyses, and writing.

## Data Availability

The data that support the findings of this study are available from the corresponding author, [SG], upon reasonable request.
